# The high explosives & affected targets (HEAT) dataset

**DOI:** 10.1016/j.dib.2026.113012

**Published:** 2026-06-22

**Authors:** B. Kaiser, K. Hickmann, S. Chakrabarti, S. De, S. Pandit, D. Schodt, J. Pulido, D. Banesh, C. Sweeney

**Affiliations:** Los Alamos National Laboratory, USA

**Keywords:** High explosives, Equations of state, Material phase change, Fluid dynamics, Shock physics, Detonation, Plastic deformation, Continuum mechanics

## Abstract

Artificial Intelligence (AI) surrogate models offer a computationally efficient alternative to full-physics simulations, yet no existing datasets are publicly available for training, testing, and validation of machine learning models of the dynamics of high-explosive driven shocks through multiple materials. Shock propagation through materials is a computationally challenging problem because simulations must include material-specific equations of state (EOS) along with descriptions of other physical processes such as plastic deformation, phase change, damage processes, fluid instabilities, and multi-material interactions. Shocks are typically initiated by high-velocity impacts or explosive loading. The latter case necessitates the addition of models of reactive materials to represent high-explosive (HE) detonation.

To address the lack of an expansive dataset for multi-material shock propagation in the AI/ML community, we present the High-Explosives and Affected Targets (HEAT) Dataset. HEAT is a physics-rich collection of two-dimensional, cylindrically symmetric, simulations generated using an Eulerian, multi-material, shock-propagation code developed at Los Alamos National Laboratory. The dataset includes two partitions: (1) the expanding shock-cylinder (CYL) simulations, Figs. 1, and (2) the Perturbed Layered Interface (PLI) simulations, Fig. 2. Entries in both partitions consist of time series of arrays of thermodynamic fields (pressure, density, and temperature), kinematic fields (position and velocity), and additional fields that depend on thermodynamic and/or kinematic fields (e.g., material stress). Materials in the CYL partition include solids (aluminium, copper, depleted uranium, stainless steel, tantalum, and a generic polymer), a liquid (water), gases (air, nitrogen), and a generic detonating material (high explosive, HE). The PLI partition spans a highly varying geometry but consists of fixed materials across entries: Copper, aluminium, stainless steel, generic polymer, and generic HE. HEAT captures critical phenomena such as momentum transfer, shock propagation, plastic deformation, and thermal effects, making HEAT a valuable benchmark for development of AI/ML emulation of multi-material shock propagation.

Specifications Table

**Table:** 

Subject	Engineering & Materials science
Specific subject area	Temporal evolution of shocks, phase change, and momentum transfer in solids, liquids, and gases subjected to high explosive detonation.
Type of data	The HEAT dataset contains 5300 Perturbed Layered Interface (PLI) simulations each sampled at 101 evenly spaced timestamps and 2161 expanding shock-cylinder (CYL) simulations each sampled at 57 evenly spaced timestamps. Thus, the HEAT dataset contains a combined total of 661,507 snapshots of Pagosa simulations, with 7,491 of those snapshots the initial conditions. Each simulation is time series of 2D kinematic and thermodynamic variable fields generated by LANL’s hydrodynamics code Pagosa. The field data is stored as arrays within .npz files (python numpy). Accordingly, there is a single .npz file containing the simulation-state arrays at each time and together many times form the time series output from a single simulation. The time series intervals (“time steps”) are constant: The data for every simulation has the same time step size and time step size is constant across the period of each simulation. Simulation units are centimeters, grams, microseconds, and Kelvin for length, mass, time, and temperature, respectively. The data is Image, Filtered.
Data collection	7491 Pagosa simulations were performed on LANL High Performance Computing (HPC) clusters. The simulation output was temporally filtered to collect evolution of 2D hydrodynamic fields (velocity, density, internal energy, pressure, stress, strain) only at pre-specified evenly spaced time intervals. The data are not normalized.
Data source location	The data is available for direct download at https://oceans11.lanl.gov/heat/
Data accessibility	Repository name: The High Explosives & Affected Targets (HEAT) Dataset Data identification number: LA-UR-25-26490 Direct URL to data: https://oceans11.lanl.gov/heat/ Users can choose to download the entire suite of PLI or CYL data by downloading the .tar files at https://oceans11.lanl.gov/heat/pli/ or https://oceans11.lanl.gov/heat/cyl/, respectively. The user may download the entire double precision PLI dataset (2.6 TB) at https://oceans11.lanl.gov/heat/pli/pli240420.tar. The user may download the double precision CYL data (183 GB) or single precision CYL data (51 GB), at https://oceans11.lanl.gov/heat/cyl/cx241203_fp16_full.tar or https://oceans11.lanl.gov/heat/cyl/cx241203_fp16_half.tar, respectively. Users may also download individual simulations for each dataset from the folders https://oceans11.lanl.gov/heat/pli/pli240420_fp16_full, https://oceans11.lanl.gov/heat/pli/pli240420_fp16_half, https://oceans11.lanl.gov/heat/cyl/cx241203_fp16_full, and https://oceans11.lanl.gov/heat/cyl/cx241203_fp16_half. Simulation number is given by id{SIM_NUMBER} and time step number is given by idx{TIME_NUMBER}, such that the first time step of the first CYL simulation is cx241203_id00001_pvi_idx00000.npz (simulations start a 1, time steps start a 0).
Related research article	None

## 1 Value of the Data

• High explosives simulations are rich multi-physics models (e.g., detonation chemistry, fluid-structure interaction, and heat transfer), giving a more comprehensive understanding than what is possible with analytical formulas (which are typically single-physics) alone. Multi-physics simulations couple conservation laws with Equations of State (EoS) for different materials along with different models for multiple phases of each material.
• The HEAT dataset's large simulation count, wide variation in material combinations, and rich multi-field outputs make it well suited for training data-driven models of multi-material shock dynamics.
• The diversity of geometries and material configurations across the HEAT dataset simulations provides a library of physically consistent states that could serve as initial conditions for downstream simulations or for sensitivity studies.
• The HEAT dataset could be used for data-driven or hybrid approaches to EoS parameter estimation and uncertainty quantification because the simulations span a wide range of pressures and strain rates across multiple materials.
• Eulerian constant-time-step, simulation-derived high explosive datasets are useful because they record the evolution of all the kinematic and thermodynamic variables on a regular grid at evenly spaced points in time.
• Simulation-derived datasets can reduce the need for experiments during early design stages of new high explosive configurations, allowing only the most promising designs to be tested physically.
• These data are valuable because real-world high explosive testing is dangerous**.** High explosive configurations pose risks to personnel and equipment.
• These data are valuable because of the high cost of real-world experiments**.** Experiments can be expensive due to the materials, safety precautions, and instrumentation required.
• Training a predictive surrogate model on these data is a process that closely resembles video generation because these data are 2D with uniform spatial and temporal discretization. Therefore, these data may also be valuable for creative video generation.

## 2 Background

AI surrogate models of conventional multi-physics and multi-material models promise computationally efficient alternatives to conventional models with similar accuracy [1]. Here, a conventional model is a numerical model that solves discretized systems of partial differential equations that describe physical systems at continuum scales under various assumptions. Analytical solutions are generally infeasible except for limited cases [2], so numerical methods that solve discretized equations with known convergence and accuracy properties are typically used. Typically, these numerical methods are computationally expensive when high accuracy is needed and computationally cheaper, lower resolution implementations tend to alias critical dynamics. The promise of fast yet accurate models motivate the development of AI surrogate models that capture only the essential features of conventional models at the relevant scale.

AI surrogate models of conventional multi-physics and multi-material models need diverse training data to forecast the evolution of coupled nonlinear dynamical systems [3]. Multi-physics AI surrogate models [4] typically approximate solutions to autoregressive prediction problems in which model f is trained to predict

$$U\left(x,t_{n+1}\right)=f\left(U\left(x,t_{n}\right)\right),$$

where U is a state vector, x are spatial coordinates, t is time, and subscript n is the time step index such that $t\in \{t_{0},t_{1},\ldots ,t_{T}\}$. AI surrogate model training data must be representative of many physical regimes to learn the functionality of conventional multi-physics models, to avoid overfitting, and to support generalization beyond the training data. To the author’s knowledge, no other publicly available dataset captures the interacting dynamics of myriad solids (aluminium, copper, depleted uranium, stainless steel, tantalum, and a generic polymer), liquids (water), gases (air, nitrogen), and detonator material (high explosive, HE) under shock loading and explosion. HEAT [5] adds a rich menagerie of new physical phenomena to support the training of surrogate models.

## 3 Data Description

The PAGOSA hydrocode was used to generate a comprehensive suite of PLI simulations, consisting of roughly 5330 individual simulations each sampled at 101 evenly spaced timestamps (the first snapshot contains the initial conditions) for a total of 538,330 snapshots, and 2161 CYL simulations each sampled at 57 evenly spaced timestamps for a total of 123,177 snapshots. Thus, the HEAT dataset contains a combined total of 661,507 snapshots of PAGOSA simulation dynamics, with 7,491 of those snapshots the initial conditions. PAGOSA simulation units are centimeters, grams, microseconds, and Kelvin for length, mass, time, and temperature, respectively. The PLI and CYL simulations were computed with single precision.

### 3.1 Perturbed layered interface (PLI) simulations

Each PLI simulation predicts the evolution of the high explosive (HE), stainless steel case, polymer cushion layer, Aluminum striker layer, Copper throw layer, and air in the configuration shown in Fig. 1 after the high explosive detonates at t = 0. Fig. 2 shows the evolution of PLI simulation id00015. Time advances in increments of roughly 0.25 microseconds, reaching a final simulated time of t = 25.0 microseconds. For every snapshot the code outputs a Eulerian grid of the full hydrodynamic field, including the (R, Z) velocity components and the (R, Z) spatial coordinates. In each grid cell the properties of every material present are recorded in .npz file arrays for every time step and every material: density, temperature, pressure, volume fraction, stress, strain, shear modulus, yield strength, plastic strain (PLST), cell‑average yield (Yield), and sound speed. The only degrees of freedom that differ from run to run are the six spline coefficients that define the four interface‑shape spline functions: *sa* (striker anchor), *st* (striker thickness), *ct* (cushion thickness), and *tt* (throw thickness). These four lines define the interfaces between the polymer cushion and Aluminum striker, between the Aluminum striker and the Copper throw, between the high explosive and the polymer cushion (density 0.892 g/cm^3^), and between the Copper throw and air, respectively.

**Fig. 1:**
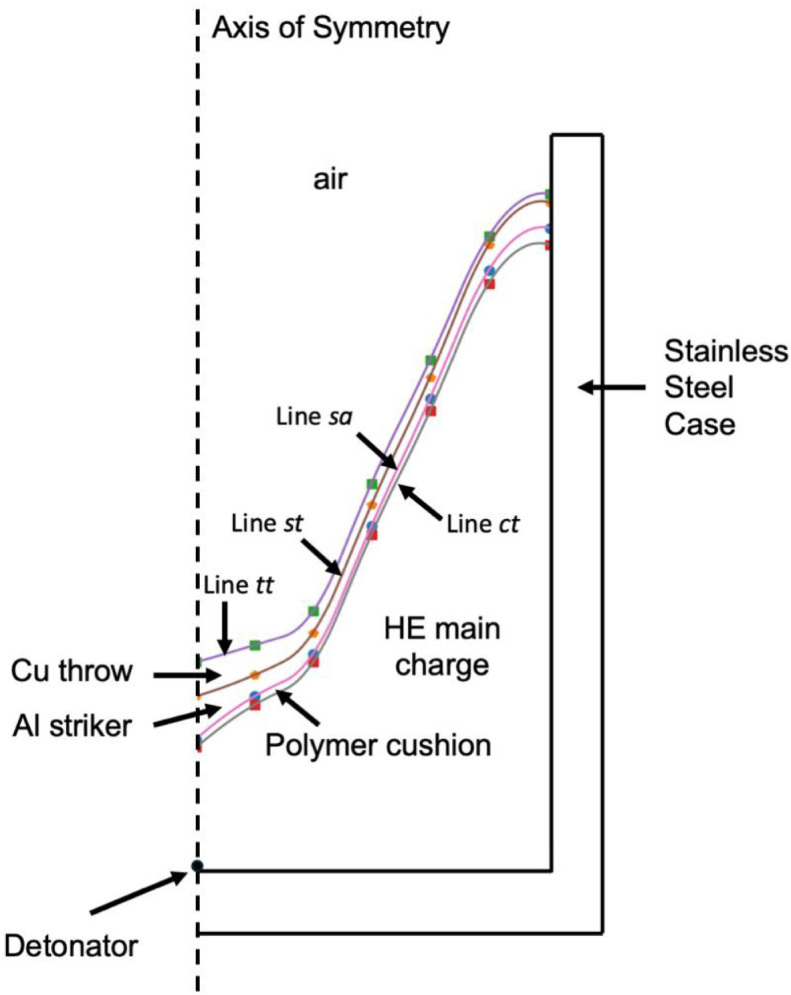
An example of the Perturbed Layered Interface at t=0. The 5330 simulations are composed of 5330 different liner geometries defined by the lines ct, sa, st, and tt, all other material and geometric parameters are held constant. Each simulation contains 100 different snapshots in time with an approximately 0.25 microsecond time step between them. At t=0 the detonator detonates and the HE burn begins.

**Fig. 2:**
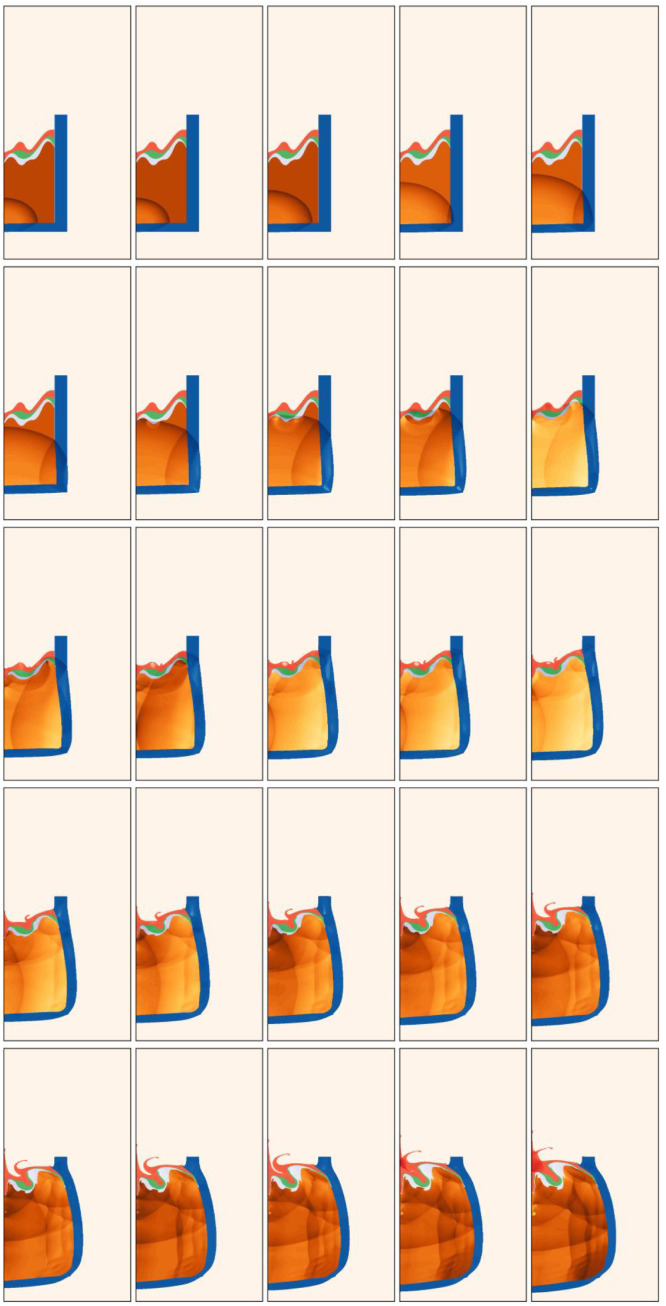
The evolution of PLI simulation id00015. Note the complex baroclinic jet geometry of the polymer cushion, Al striker, and Cu throw that forms around the axis of symmetry, the plastic deformation of the stainless steel case, and the reflected shock waves within the high explosive.

The coefficients of the spline functions for each simulation at t=0 are listed in the catalogue pli.csv hosted in /heat/pli/ on oceans11.lanl.gov [5]. The simulations (listed as id00001, id00002, etc) can be downloaded individually in /heat/pli/pli240420. Within each simulation directory individual timesteps may be downloaded. For example, /heat/pli/pli240420/id00001 contains 101 individual .npz files that each contain the hydrodynamic data at one snapshot in time. The entire dataset may be downloaded at once by downloading /heat/pli/pli240420.tar. The data sets on oceans11.lanl.gov with suffix ``_full'' and ``_half'' refers to the average “full” resolution of 250 by 250 micrometers and half that resolution, respectively. The minimum and maximum of the full resolution are 234 by 156 micrometers and 313 by 313 micrometers. The domain size is 10 cm in the radial direction r (horizontal in Fig. 1) and 28 cm in the z direction (vertical in Fig. 1). The detonator is located at (r,z) = (0,0) for every PLI simulation.

### 3.2 Expanding shock-cylinder (CYL) simulations

Each simulation in the cylex collection represents a 2-D axis-symmetric configuration: a tube of high-explosive (HE) is encased by a cylindrical wall, which in turn is surrounded by a background material (Fig. 3). Fig. 4 shows the evolution of CYL simulation id00433, in which the high explosive is surrounded by an Aluminum wall and air background. The thickness of the HE, the thickness of the cylinder surrounding the HE, and the detonator location are all randomly varied. The energy source consists of two HMX-derived HE components (a booster and a main charge) with prescribed burn rates. The wall and background materials are varied for nearly all possible combinations of materials, drawing from six strength-modelled materials (Cu, stainless steel, Al, Be, depleted-uranium, Ta) and four strength-less media (air, water, nitrogen, Sylgard polymer) in their pre-shock phases. Physical processes captured include momentum transfer, HE‑driven momentum divergence/energy input, heat transfer, shock propagation, compressible flow, and plastic flow.

**Fig. 3:**
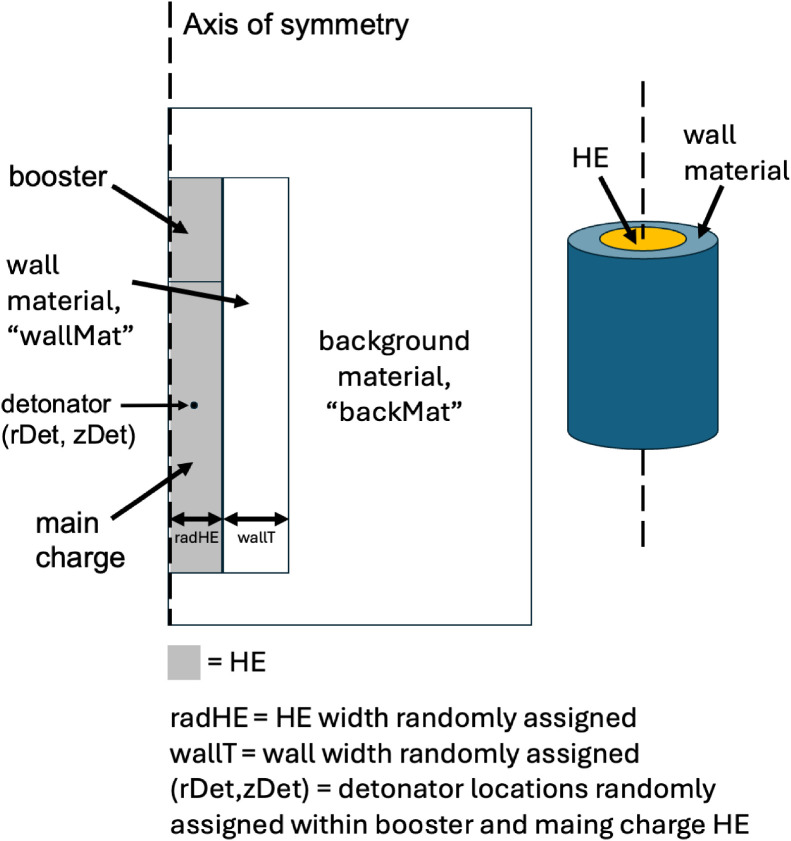
An example of the Expanding Shock-Cylinder at t=0. The detonation location (rDet,zDet), HE width (radHE), cylinder wall thickness (wallT), wall material, and background material are all varied. Each simulation contains 57 different snapshots in time with an approximately 0.25 microsecond time step between them. At t=0 the detonator detonates and the HE burn begins.

**Fig. 4:**
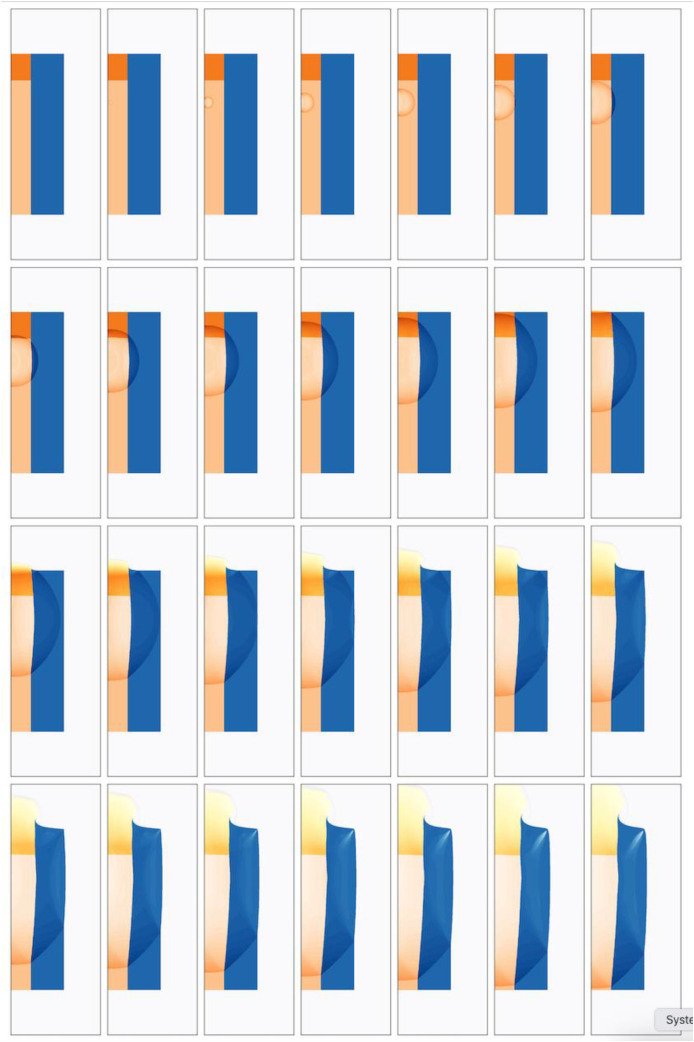
The evolution of CYL simulation id00433. The detonation in the upper part of the high explosive near the axis of symmetry causes the high explosive booster to move outward into the air background, while shock waves within the aluminum cylinder wall create plastic deformation along the upper surface of the aluminum cylinder.

For every CYL snapshot (.npz file) the following fields are stored: Density, pressure, internal energy, and volume‑fraction fields for each material (3–4 material density fields per simulation), velocity and position fields. Within oceans11.lanl.gov/heat/cyl is cyl.csv, which contains the key necessary for understanding the arrays in the npz files for each CYL simulation. For example, cyl.csv shows that the simulation cx241202_id00320 was of a cylinder made of Aluminum (wallMat) surrounded by Tantalum (backMat). In that simulation, the high explosive detonator was placed at the r,z (rDet, zDet) location 0.211, -2.889 and the radius of the high explosive (radHE) was 0.665 (cm) while the Aluminum cylinder wall thickness (wallT) was 4.608 (cm). The simulations (listed as id00001, id00002, etc) can be downloaded individually in /heat/cyl/cx241202. Within each simulation directory individual timesteps may be downloaded. For example, /heat/ cyl/cx241202/id00001 contains 57 individual .npz files that each contain the hydrodynamic data at one snapshot in time. The entire dataset may be downloaded at once by downloading /heat/ cyl/cx241202.tar. The data sets on oceans11.lanl.gov with suffix ``_full'' and ``_half'' refers to the average “full” resolution of 250 by 250 micrometers and half that resolution, respectively. The minimum and maximum of the full resolution are 234 by 156 micrometers and 313 by 313 micrometers. The domain size is 10 cm in the radial direction r (horizontal in Fig. 3) and 28 cm in the z direction (vertical in Fig. 3).

## 4 Experimental Design, Materials and Methods

Both the CYL and PLI sub-datasets were generated using the PAGOSA code of Los Alamos National Laboratory (LANL). PAGOSA is a computational fluid dynamics code developed for simulating high-speed compressible flows and high-rate material deformation [6]. It is a Eulerian reference frame finite-difference code designed to handle complex interactions among gases, fluids, and solids, with broad support for diverse equations of state (EOS), material strength models, and explosive behavior.

PAGOSA is particularly suited for studying extreme physical phenomena such as explosively driven systems, high-velocity impacts, and other high-pressure, high strain-rate events, where pressures range from kilobars to megabars. In this regime, materials undergo significant volume changes, making incompressibility assumptions invalid. The code is intended to resolve the propagation of compression and rarefaction waves within materials—critical for accurately modeling dynamic events in continuum mechanics. Accordingly, PAGOSA has been used to simulate shaped charges [7], explosively fired projectiles [8], and other high explosive tests for proton radiography [9].

High explosive detonation was modeled using the FSD (Fast Sweeping Detonation) burn model is an alternative programmed burn method which calculates burn times by solving the Eikonal equation using the Fast Sweeping Method [6]. The FSD burn model was computed with second order spatial accuracy. A recent validation study of CYL-like geometries has shown that the grid resolutions used for the CYL and PLI simulations (both approximately 250 micrometers uniform) agree well with experimental Photonic Doppler Velocimetry data for program burn [10], the detonation model used in both PLI and CYL datasets.

The timestep in the simulations was varied to remain smaller than that given by the Courant-Friedrichs-Lewy (CFL) condition for the bulk material velocity and the shock velocity. The Courant number (i.e., safety factor) for the two velocities was set to 0.25 and 0.75, respectively. The time step was allowed to grow by increase is 5% in step size from cycle to cycle so long as the CLF conditions were satisfied and the time step was not allowed to be larger than 10 microseconds. The initial time step was specified as the minimum time step size, 1e-6 microseconds. The HEAT data set temporal snapshots do not include all of the computed time steps but rather just the time steps closest to a 0.25 microsecond interval.

The boundary conditions were reflective, rigid, no (zero) flow across the boundaries on all four sides for every PLI and CYL simulation.

The PLI geometry (Fig. 1) features three nested metallic layers (Al striker, Cu throw, and polymer cushion) whose interfaces are defined using parametric contours. Each interface contour is constructed through a two-stage process: (1) generating anchor points along a parabolic base curve, and (2) applying B-spline interpolation to create smooth contours. These anchor points would then be used to create a spline interpolation elsewhere. The z-coordinates of the anchor points are determined by

$$z\left(r\right)=\left(\frac{h_{w}-h_{c}}{r_{w}^{2}}\right)r^{2}+h_{c}\;\text{cm},$$

where *r* is the radial coordinate in cm, *h_w_* is the height of the parabola at the case wall (fixed at 9.0 cm), *h_c_* is the height of the parabola at the center (varies from 4.0 to 9.0 cm), and *r_w_* is the radius of the case wall (fixed at 4.0 cm). The parabola achieves its minimum at the centerline (r = 0) with height *h_c_* and reaches height *h_w_* at the wall radius *r_w_.* This function is evaluated at seven fixed radial anchor positions**:**

$$r_{anchors}=\left[0.0,\;0.66,\;1.32,\;1.98,\;2.64,\;3.3,\;4.0\right]\;\text{cm},$$

These positions are uniformly spaced at 0.66 cm intervals from the centerline to the inner case wall. The endpoint values (0.0 and 4.0 cm) are hard-coded to ensure the geometry properly spans from the axis of symmetry to the case wall boundary.

There are 10,000 total geometry variations: 20 base geometries × 500 perturbations each**.** The base parabola profiles are twenty distinct parabolic curvatures are generated by evaluating the expression for z(r) above while uniformly varying *h_c_* from 4.0 to 9.0 cm with spacings of 0.263 cm between each of the 20 *h_c_* values. For each base parabola profile, 500 perturbed versions are created by adding random variations:

$$z_{striker}^{i,j}=\;z\left(r_{anchors}^{j};\;h_{c}^{i}\right)+\epsilon^{i,j},$$

$$\epsilon^{i,j}∼\;\text{Uniform}\left(-1.0,\;1.0\right)\;\text{cm},$$

i∈{1,…,10000},

j∈{1,…,7},

where i divided by 500 determines which base parabola is used. Note that *z_striker_* is the lower surface of the striker, equivalent to line *sa* in Fig. 1. The layer thicknesses of the Al striker, Cu throw, and Sylgard (polymer) cushion layers at each anchor point are independently sampled from uniform distributions

$$t_{striker}^{i,j}=z_{st}-z_{striker}\;∼\;\text{Uniform}\left(0.05,1.0\right)\;\text{cm},$$

$$t_{throw}^{i,j}=z_{tt}-z_{st}\;∼\;\text{Uniform}\left(0.05,1.0\right)\;\text{cm},$$

$$t_{cushion}^{i,j}=z_{striker}-z_{ct}\;∼\;\text{Uniform}\left(0.05,1.0\right)\;\text{cm},$$

respectively, where *z_sa_* corresponds to line *sa* in Fig. 1, *z_st_* corresponds to line *st* in Fig. 1, *z_ct_* corresponds to line *ct* in Fig. 1, and *z_tt_* corresponds to line *tt* in Fig. 1. The minimum thickness (0.05 cm) ensures physical realizability while the maximum (1.0 cm) provides substantial geometric variation.

The seven anchor points for each layer (such as the points at $r_{anchors}$, $z_{striker}$ for the lower surface of the striker) are interpolated using SciPy v1.17.0′s B-spline implementation [11] with *k*=2 (quadratic spline) and smoothing parameter *s* = 0.0 (interpolating spline that passes exactly through all anchor points). This produces smooth, continuous contours for the striker back surface and each layer interface throughout the radial domain [0, 4.0] cm.

The CYL simulations (Fig. 3, Fig. 4) employ a systematic random sampling approach to generate 2,660 distinct configurations, combining 133 unique material pairings with 20 independently sampled geometry configurations each. The material sampling strategy entailed 133 combinations of wall and background materials selected from a palette of 12 options: Void, aluminum, beryllium, depleted uranium, Sylgard polymer, tantalum, 304L stainless steel, tin, copper, nitrogen, air, and water (Table 1). The sampling followed a hierarchical structure with physical constraints: one special case pairs Void wall with Void background, 11 combinations pair each non-Void material with a Void background, and the remaining 121 combinations represent all possible non-Void wall and background pairings. A constraint enforces that if the wall material is Void, the background material must also be Void, preventing physically nonsensical configurations. For materials requiring tabulated equation-of-state (EOS) data, the system initializes each to zero pressure using SOPES queries of SESAME tables, with reference densities ranging from 0.9998 g/cm³ for water to 19.043 g/cm³ for depleted uranium. Nitrogen uses an ideal-gas EOS, while air is excluded from zero-pressure initialization to avoid extrapolation errors beyond the SESAME table boundaries. The PLI simulations use the same thermodynamic constitutive models as the CYL simulations.

**Table 1:** Initial (pre-shock) densities for the simulated materials.

Material	Initial Density (g/cm³)
Air	1.293e-3
Al	2.712
Cu	8.940
Depleted U	19.043
N_2_	1.251e-3
Sn	7.290
Stainless Steel (304L)	7.910
Sylgard	1.037
Ta	16.681
Void	0.0
Water	1.0

The CYL simulation geometric parameters follow a uniform random sampling scheme with hierarchical dependencies ensuring physical realizability. The high-explosive (HE) radius samples uniformly from [0.25, 4.0] cm, establishing the fundamental length scale for each configuration. Two parameters depend conditionally on this HE radius: wall thickness samples uniformly from [0.1, 6.0 - radHE] cm to ensure the outer wall radius never exceeds the 6.0 cm mesh dimension limit. Random numbers were generated using NumPy's default_rng random number generator initialized with seed 255541. This creates a constrained quasi-random design where the two independent parameters (material combinations, HE radius) establish the framework, while two dependent parameters (wall thickness and radial detector position) adapt to respect geometric constraints, effectively producing a space-filling design that systematically explores feasible configurations for the detonation physics problem.

The initial pressure, density, and internal energy were specified for each material using the SESAME Equations of State [12] except for N_2_ and air (ideal gas EOS) as well as Aluminum, depleted Uranium, Tantalum, and Sylgard. The initial density was specified as the standard temperature and pressure reference value for each material, then the Equations of State were used to compute the specific internal energy constrained such that at time t=0 pressure is zero everywhere in the domain (i.e., no pressure gradients at t=0). The SESAME EOS tables for each applicable material are those described in [12].

The largest characteristic Mach numbers for the shock front (the ratio of the magnitude of the simulated material velocity divided by the sound speed assigned to each material) in the PLI and CYL simulations are roughly 25 and they occur for air and N_2_. The largest Mach number regime for the shock front in the other materials (the metals, polymer, Sylgard) range from roughly 1.4 to 2.6. These shock front Mach numbers are upper bounds for the initially solid materials because speed of sound generally decreases with temperature in these materials and the materials warm upon shock. Maximum simulated pressures are order 10 GPa.

The material strength model is the Standard Strength Model. The Standard Strength Model is an isotropic strength model and can use any of the flow-stress models (also known as yield strength), which are discussed below. Also, this model can use any one of the fracture models listed below in this manual. This is an isostropic elastoplastic strength model with radial return of the yield surface. By default, it uses the Jaumann stress rate that is objective with respect to rigid body rotation and translation. A material is allowed to undergo an elastoplastic transition. The yield criterion determines the transitional point at which material stops deforming elastically and begins to deform plastically. The model uses the von Mises (radial return) criteria to get that point. Then the stress deviators are adjusted so they will remain on the yield surface during plastic flow. The flow-stress model calculates the yield strength and shear modulus. No damage models were used to model material fracture.

After a material can no longer sustain any strength (generally due to a change of phase to continuously deformable fluid). In this case, PAGOSA solves a discrete form of the hydrodynamic equations [6]:

$$\frac{\partial \rho \;}{\partial t}+u\frac{\partial \rho \;}{\partial x}+v\frac{\partial \rho \;}{\partial y}=-\rho \;\left(\frac{\partial u}{\partial x}+\frac{\partial v}{\partial y}\right),$$

$$\frac{\partial u}{\partial t}+u\frac{\partial u}{\partial x}+v\frac{\partial u}{\partial y}=-\frac{1}{\rho }\frac{\partial p}{\partial x}+\frac{F_{x}}{\rho }+\frac{1}{\rho }\left(\frac{\partial S_{\text{xx}}}{\partial x}+\frac{\partial S_{\text{xy}}}{\partial y}\right),$$

$$\frac{\partial v}{\partial t}+u\frac{\partial v}{\partial x}+v\frac{\partial v}{\partial y}=-\frac{1}{\rho }\frac{\partial p}{\partial y}+\frac{F_{y}}{\rho }+\frac{1}{\rho }\left(\frac{\partial S_{\text{yx}}}{\partial x}+\frac{\partial S_{\text{yy}}}{\partial y}\right),$$

$$\frac{\partial e}{\partial t}+u\frac{\partial e}{\partial x}+v\frac{\partial e}{\partial y}=-\frac{p}{\rho }\;\left(\frac{\partial u}{\partial x}+\frac{\partial v}{\partial y}\right)+\frac{1}{\rho }\left(S_{\text{xx}}e_{xx}+S_{\text{yy}}e_{yy}+2S_{\text{xy}}e_{xy}\right),$$

where the strain rate tensor elements are

$$e_{xx}=\frac{\partial u}{\partial x},$$

$$e_{yy}=\frac{\partial v}{\partial y},$$

$$e_{xy}=\frac{1}{2}\left(\frac{\partial u}{\partial y}+\frac{\partial v}{\partial x}\right),$$

and the deviatoric stress tensor elements are

$$S_{\text{xx}}=2G\left(e_{xx}-\frac{1}{3}\left(\frac{\partial u}{\partial x}+\frac{\partial v}{\partial y}\right)\right),$$

$$S_{\text{yy}}=2G\left(e_{yy}-\frac{1}{3}\left(\frac{\partial u}{\partial x}+\frac{\partial v}{\partial y}\right)\right),$$

$$S_{\text{xy}}=2Ge_{xy},$$

where *G* is the shear modulus. An artificial viscosity model is required to stabilize the simulations and accurately capture shock waves without introducing nonphysical oscillations. We use the Wilkins model [6] for artificial viscosity.

## Limitations

None of the materials in the PLI and CYL data included a damage model for solids subjected to shock loading. The simulated solids can only deform elastically and plastically and never break, crack, or spall, where spall is defined as tensile fractures that arise from reflected shock wave patterns within a solid. Therefore, the solid dynamics captured in HEAT overpredict load-carrying capacity and survivability because shocks are absorbed that may otherwise cause fractures and spallation. This shock absorption can cause secondary inaccuracies due to unphysical temperature increases, plastic work, and wave damping.

Conventional models that use a Eulerian hydrodynamic reference frame, such as Pagosa, model fluid motion by solving the governing equations on a fixed spatial grid through which the fluid flows. These discrete models have many sources and types of errors [4]. For example, as the materials move through the grid sharp interfaces (e.g., between metal and air) can become blurred over time due to numerical diffusion. This can cause unphysical states, such as metal-air interface cells with unrealistic densities or pressures. Flux calculation numerical errors can lead to small violations of conservation laws that steadily accumulate as the forecast progresses in time. Interface tracking numerical errors that occur in mixed-material cells can also cause cumulative errors in global quantities and unphysical heating or mass loss/gain. Fixed spatial grids can also impose artificial directional bias. Finally, different materials have different Equations of State (EOS) that are sources of epistemic error [4], which arise from a lack of understanding of the underlying physical processes. Weighted averages of EOS values in interface cells can create thermodynamic inconsistencies. Long term forecasts of coupled and highly nonlinear systems generate aleatoric errors [4], which arise from intrinsic randomness. Together, these factors highlight how both epistemic and aleatoric uncertainties, compounded by numerical and modelling errors inherent to Eulerian formulations, can significantly degrade the physical fidelity and predictive reliability of conventional hydrodynamic simulations.

## Ethics Statement

The authors have read and follow the ethical requirements for publication in Data in Brief and confirming that the current work does not involve human subjects, animal experiments, or any data collected from social media platforms.

## Credit Author Statement

**Bryan Kaiser**: Data Curation, Writing, Original draft preparation, Editing, Software, Methodology. **Kyle Hickmann**: Conceptualization, Software, Methodology, Editing, Supervision. **Sharmistha Chakrabarti**: Software, Methodology. **Soumi De:** Software, Methodology. **Sourabh Pandit**: Data Curation, Software. **David Schodt**: Software. **Jesus Pulido**: Data Curation. **Divya Banesh**: Data Curation. **Christine Sweeney:** Data Curation.

## Data Availability

oceans11.lanl.govThe High Explosives and Affected Targets (HEAT) Dataset (Original data).

## Appendix Supplementary materials

Image, application 1
