# Supplementary material for: The high explosives & affected targets (HEAT) dataset
Source: Data Brief. 2026 Jun 22;67:113012. doi: 10.1016/j.dib.2026.113012 (PMC13320550; doi:10.1016/j.dib.2026.113012)
Supplement: Supplementary file 1 [file mmc1.pdf]

ELSEVIER

Authorship change request

Important information – please read before continuing.

How to use this form

- This form is to be completed by the Corresponding Author to request any change in authorship (additions, removals, or reordering) after the submission of a manuscript, including changes in Corresponding Authors, if any.
- Changes to the author list must not be made in the journal's **Editorial Manager** system without submitting this form. Unauthorized authorship changes will result in the rejection of your submission, or retraction if the article has already been published.
- Authorship changes are not allowed after manuscript acceptance. It is also not possible to submit this form after your manuscript has been rejected, for any reason.
- Do not use this form for name changes or corrections.
- This form consists of three parts, all of which should be completed prior to submission.

A note about author disputes

The publisher and editor cannot investigate or mediate any authorship disputes.

If you are unable to obtain agreement from all authors, including those you intend to remove, we recommend seeking guidance from your institution. We will not consider your change request and will not proceed with the publication of your manuscript until all outstanding authorship disputes are resolved.

Before completing the form

- All authors should carefully review the "Duties of Authors" section of the Elsevier publishing ethics policy, particularly the sections on:
    - Authorship of the paper
    - The use of generative AI and AI-assisted technologies in scientific writing and in figures, images and artwork
- Please also carefully review the submission journal's "Guide for authors" (this might also be referred to as "Instructions for authors"), because some journals may have additional authorship criteria (an example is the ICMJE guidelines for authorship).
- Be prepared to enter the full name, email address, and institution for every author on the manuscript. This information must match the author list in the submission system.

How to submit this form

After completing this form according to the above instructions, it should be submitted through the journal's **Editorial Manager** system with your revised manuscript via the "Submissions Needing Revision" link.

(*continued from the previous page*)

In the “Attach Files” step, use the “Cover letter” item type and in the description, type “Authorship change request form” and upload this form. Make corresponding changes in the Manuscript Data step before completing your revision submission.

For additional guidance, view the tutorial on using Editorial Manager for this process in the Journal Publishing Support Center.

Once submitted, the information in this form will become part of the revision submission record.

Part 1. General submission information

To be completed by **the Corresponding Author**.

Manuscript details

Journal title	Data In Brief
Manuscript and/ or article number	DIB-D-26-00786
Manuscript title	The High Explosives & Affected Targets (HEAT) Dataset.

Change(s) requested (check all that apply)

☑ Add new author(s)	☐ Remove author(s)
☐ Change the Corresponding Author	☐ Change the order of authors*

* If ONLY changing order of existing authors, go directly to Part 3. Do not complete Part 2.

Part 2. Indicate author(s) to be added or removed

For each author to be added or removed complete one template below.**

In the "**Reason for this change**" section, please include as much detail as possible so we can evaluate if the change is approved. At a minimum, this should include explanations for both:

- why the change is being requested, and
- why the author was/was not included in the original author list.

Important Note: If this part is either not provided, incomplete, or the reasons provide insufficient detail or do not address the points above, your request will be denied, and your submission may be rejected.

2.1 Author change information

Given/first name(s)	Sourabh
Family/last name	Pandit
Email address	spandit@lanl.gov
Institution	Los Alamos National Laboratory

Change(s) requested for this author

☐ Remove author ☑ Add new author ☐ Make Corresponding Author

Author's Individual contributions (required for author additions only) see CRediT Contributor Roles Taxonomy

☐ Conceptualization	☑ Data curation	☐ Formal analysis
☐ Funding acquisition	☐ Investigation	☐ Methodology
☐ Project administration	☐ Resources	☑ Software
☐ Supervision	☐ Validation	☐ Visualization
☐ Writing – original draft	☐ Writing – review & editing	

Reason for this change

SP assisted in creating the data loader for the PLI portion of the dataset and helped identify corrupted files. Not including SP on the original author list was an oversight due to contributions occurring significantly prior to manuscript preparation.

2.2 Author change information

Given/first name(s)	David
Family/last name	Schodt
Email address	dschodt@lanl.gov
Institution	Los Alamos National Laboratory

Change(s) requested for this author ☐ Remove author ☑ Add new author ☐ Make Corresponding Author

Author's Individual contributions (required for author additions only)
see CRediT Contributor Roles Taxonomy

☐ Conceptualization	☐ Data curation	☐ Formal analysis
☐ Funding acquisition	☐ Investigation	☐ Methodology
☐ Project administration	☐ Resources	☑ Software
☐ Supervision	☐ Validation	☐ Visualization
☐ Writing – original draft	☐ Writing – review & editing	

Reason for this change

DS assisted in creating the data loader for the PLI portion of the dataset. Not including DS on the original author list was an oversight due to contributions occurring significantly prior to manuscript preparation.

2.3 Author change information

Given/first name(s)	
Family/last name	
Email address	@
Institution	

Change(s) requested for this author ☐ Remove author ☐ Add new author ☐ Make Corresponding Author

Author's Individual contributions (required for author additions only) see CRediT Contributor Roles Taxonomy

☐ Conceptualization	☐ Data curation	☐ Formal analysis
☐ Funding acquisition	☐ Investigation	☐ Methodology
☐ Project administration	☐ Resources	☐ Software
☐ Supervision	☐ Validation	☐ Visualization
☐ Writing – original draft	☐ Writing – review & editing	

Reason for this change

2.4 Author change information

Given/first name(s)	
Family/last name	
Email address	@
Institution	

Change(s) requested for this author ☐ Remove author ☐ Add new author ☐ Make Corresponding Author

Author's Individual contributions (required for author additions only) see [CRediT Contributor Roles Taxonomy]

☐ Conceptualization	☐ Data curation	☐ Formal analysis
☐ Funding acquisition	☐ Investigation	☐ Methodology
☐ Project administration	☐ Resources	☐ Software
☐ Supervision	☐ Validation	☐ Visualization
☐ Writing – original draft	☐ Writing – review & editing	

Reason for this change

2.5 Author change information

Given/first name(s)	
Family/last name	
Email address	@
Institution	

Change(s) requested for this author ☐ Remove author ☐ Add new author ☐ Make Corresponding Author

Author's Individual contributions (required for author additions only) see [CRediT Contributor Roles Taxonomy]

☐ Conceptualization	☐ Data curation	☐ Formal analysis
☐ Funding acquisition	☐ Investigation	☐ Methodology
☐ Project administration	☐ Resources	☐ Software
☐ Supervision	☐ Validation	☐ Visualization
☐ Writing – original draft	☐ Writing – review & editing	

Reason for this change

** If you have more than 5 author changes, add additional pages of the above template to provide their details.

Part 3. Confirm author order and agreement

1. Listing removed and proposed authors

Please enter the full name and details for all the removed authors in the table below, "**Agreement of removed authors**."

In the next table, "**Proposed author list**," enter the full name and details of all the proposed authors *in the order that they should appear* in the publication.

"**Full name**" is the author's name as it appears in the submission system author list.

2. Gathering signatures of all authors

This form must be signed individually by every author, including both added and removed authors. The only exception is for cases where consortia group authorship was declared at submission, in which case the corresponding author may sign on behalf of the group.

While handwritten signatures are acceptable, we highly encourage the use of electronic signature software (DocuSign, Adobe Sign, Dropbox Sign, or similar) with valid e-signatures. These digital signatures should reflect your institutional information and email as provided in the author list below.

By signing this form all authors agree:

1) that they have read and acknowledge the publishing ethics policies linked in the "Important Information" section of this form, and;

2) to the addition and/or removal of the authors listed in section 2 and to the revised order of the author list in this section 3, and;

3) that all information provided accurately reflects the authorship of the article.

Agreement of removed authors

Full name	Email address	Signature	Date
	@		
	@		
	@		
	@		
	@		

Add additional page(s) if needed.

Proposed author list

Order	Full name	Email address	Signature	Date
01	Bryan Kaiser	bkaiser@lanl.gov	[illegible]	04/14/2026
02	Kyle Hickmann	hickmank@lanl.gov	Kyle Hickmann	04/14/2026
03	Sharmistha Chakrabarti	schakrabart@lanl.gov	Sharmistha Chakrabarti	04/14/2026
04	Soumi De	soumide@lanl.gov	Soumi De Digitally signed by Soumi De Date: 2026.04.20 21:29:42 -06'00'	04/15/2026
05	Sourabh Pandit	spandit@lanl.gov	S.Pandit	04/16/2026
06	David Schodt	dschodt@lanl.gov	David Schodt	04/16/2026
07	Jesus Pulido	pulido@lanl.gov	[illegible]	04/18/2026
08	Divya Banesh	dbanesh@lanl.gov	Divya Banesh	04/19/2026
09	Christine Sweeney	cahrens@lanl.gov	Christine Sweeney Digitally signed by Christine Sweeney Date: 2026.04.20 10:52:52 -06'00'	04/20/2026
10		@		
11		@		
12		@		
13		@		
14		@		
15		@		
16		@		
17		@		
18		@		
19		@		
20		@		
21		@		
22		@		
23		@		
24		@		
25		@		

Add additional page(s) if needed.
